# Insights into Catalytic Oxidative Reaction Mechanisms of Pentane on the Ru(0001) Surface

**DOI:** 10.1021/acs.jpcc.4c05735

**Published:** 2024-11-11

**Authors:** Mazharul M. Islam, C. Richard A. Catlow, Alberto Roldan

**Affiliations:** 1 Cardiff Catalysis Institute, School of Chemistry, University of Cardiff, Main Building, Park Pl, Cardiff CF10 3AT, U.K.; 2 Department of Chemistry, University College London, 20 Gordon St., London WC1 HOAJ, U.K.

## Abstract

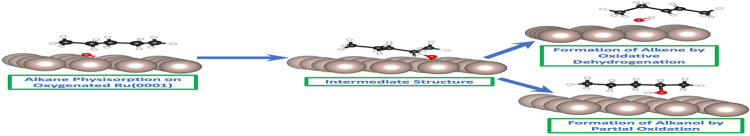

Developing efficient and selective oxidative transformations of light alkanes into alkenes or oxygenates is vital for advancing to cleaner and more efficient chemical processes. A suitable selective catalyst is required to ease reaction conditions and ensure the formation of desired oxygenated compounds. Here, using periodic density functional theory, we have investigated the suitability of a ruthenium catalyst for the partial oxidation of *n*-pentane using molecular oxygen. The first step of the process involves the dehydrogenation of primary or secondary carbons in the aliphatic chain, resulting in an adsorbed hydride structure on the metal surface. The intermediate may proceed through different reaction pathways, leading to various products. The successive dehydrogenation, a faster process than the first oxidative dehydrogenation, produces pentene and a water molecule. Alternatively, the direct interaction of the hydroxyl group with the pentyl hydride produces alcohol. The atomistic simulations reveal that Ru is a suitable candidate for catalyzing the conversion of alkanes into alkenes and oxygenates. As a significant outcome, we have observed that catalytic oxidative dehydrogenation is more feasible than direct catalytic dehydrogenation for yielding olefins from alkanes.

## 1 Introduction

The production of olefins and oxygenates from alkanes has received considerable attention in both industry and academia due to the high abundance and low cost of alkanes and the critical role of olefins as feedstocks in the production of polymers and bulk chemicals.^1,2^ Alkanes are chemically stable due to nonpolar, strong, and localized C–C and C–H bonds, requiring intensive reaction conditions to promote their reactivity.^3^ Large alkanes are converted into light olefins mainly by petroleum-derived steam cracking and fluid catalytic cracking (FCC). In contrast, the conversion of small alkanes into olefins can proceed through methanol to olefins (MTO) and Fischer–Tropsch to olefins (FTO) processes, see ref (4) and references therein. These processes often come with significant drawbacks, including high energy consumption, poor olefin yield, and unavoidable side reactions, *e.g.*, hydrogenolysis, cracking, and isomerization.^5−9^ In addition, the partially oxidized products tend to be more reactive than the parent alkanes, commonly leading to carbon dioxide instead of the target compounds.^10^ Therefore, controlling the selectivity is critical to obtain the desired products.

Alternative methods, including oxidative dehydrogenation (ODH) and O-insertion, overcome the limitations of partial alkane oxidation.^4^ A crucial step in these processes is the development of efficient catalysts that selectively activate C–C and C–H bonds. The olefin formation by oxidative dehydrogenation of alkanes is thermodynamically favorable, mainly due to water formation as a byproduct.^11^ Nearly complete conversion can be achieved even at relatively low temperatures, offering significant advantages over nonoxidative processes from engineering and economic perspectives. By carefully controlling reaction conditions such as temperature, pressure, and reactant concentrations and using a suitable catalyst, it is possible to favor the formation of syngas over the complete oxidation of products. Syngas is a mixture of CO and hydrogen and forms a feedstock for synthesizing a wide range of products, including methanol and synthetic hydrocarbon fuels.^12,13^ Alternatively, the controlled oxidation strategy offers a way to convert alkanes into oxygenates (alcohols, aldehydes, acids) without the intermediate syngas production, which provides an efficient approach for improving the sustainability and utility of alkane selective oxidation processes, enabling a shift toward greener chemical production.^4,14,15^

Designing catalysts that promote the selective formation of olefins and suitable oxygenates from saturated hydrocarbons is crucially important but highly challenging. The discovery of the V–Mg–O catalysts by Kung and co-workers in the early 1960s led to extensive research on the ODH of light alkanes.^11,16,17^ By studying the ODH of propane, isobutane, butane, pentane, and cyclohexane over Mg_3_(VO_4_)_2_–MgO and (VO)_2_P_2_O_7_ catalysts, it was shown that the product distribution depends on the nature of the alkane as well as the catalyst.^11^ The most widely used solid catalysts for partial oxidation reactions are metals, metal oxides, and metal complexes immobilized in zeolites, silica, alumina, polymeric resins,^18^ and metal–organic frameworks (MOFs).^19^ Noble metal catalysts show significant efficiency in breaking alkane C–C and C–H bonds.^20−23^ Various metals, including rhodium, platinum, nickel, iridium, and palladium, have been employed to catalyze partial oxidation reactions. Previous results with lighter alkanes have indicated that Rh catalysts exhibit superior performance for syngas production, whereas Pt yielded higher selectivity toward olefins.^24−26^ This differentiation in catalytic behavior highlights the importance of selecting appropriate catalysts for desired reaction outcomes.

In this work, we have considered a Ru catalyst to study the selective oxidation of pentane as part of a series of investigations aimed at identifying suitable metal-based catalysts for polyolefin upcycling processes. In previous investigations, we have demonstrated that Ru exhibits an appropriate capacity for activating C–C and C–H bonds, similar to the widely used Pt catalyst.^27,28^ Given that Ru is currently cheaper than Pt,^29^ it presents a potentially economically viable option for catalysis. Pentane is used as a model hydrocarbon for two main reasons: it is thermodynamically more stable than its longer homologues,^30^ and C5-alkanes are particularly suited for oxidative dehydrogenation and oxygen insertion, facilitating their conversion into feedstocks for many critical chemical production processes.^31−37^ Zhang et al. have observed that the catalytic partial oxidation of *n*-C_5_H_12_ on Fe/Al_2_O_3_ catalysts yields oxygenated products such as R–OH and R–CHO, in addition to syngas.^37^ We have performed density functional theory (DFT) based computational calculations to study the reaction energy profiles for oxidative dehydrogenation and O-insertion reactions of *n*-pentane molecule. Our results reveal new mechanistic insights into these key catalytic processes.

## 2 Models and Computational Methods

Our study of the partial oxidation of pentane (C_5_H_12_) on the hcp Ru(0001) surface has employed periodic DFT calculations using the plane-wave package VASP.^38−40^ Benchmark reports have confirmed that this surface plane is the most stable for this metal and, therefore, the most prevalent in catalysts.^21,41−43^ The calculations employ the generalized gradient approximation (GGA) based revised Perdew–Burke–Ernzerhof (RPBE) exchange-correlation functional,^44,45^ with long-range dispersion corrections including a zero-damping function.^46,47^ The core electrons for Ru, C, H, and O were defined by standard sets of pseudopotentials (PPs) within the projector-augmented wave (PAW) method.^48^ We have used a converged plane-wave energy cutoff of 520 eV. The integration in reciprocal space was performed with a Monkhorst–Pack ***k***-points grid.^49^ The grid was augmented at 14 × 14 × 8 ***k***-points for the bulk Ru to achieve 10^–6^ eV and 10^–3^ eV/Å for the electronic and ionic threshold convergence, respectively. The same method, including these technical options, has been successfully employed for successive hydrogenolysis and dehydrogenation of pentane on the Ru(0001) surface.^27,28^

Based on our previous studies’ systematic optimization of slab thickness and supercell size, we have considered a 6-layer slab with a *p*(4 × 4) supercell, where the bottom three layers were frozen at the optimized bulk lattice.^27,28^ The slab area was sufficient to minimize the lateral interactions between periodic images of adsorbates.^27,28^ A vacuum layer of 20 Å along the direction perpendicular to the surface was employed to prevent spurious interactions between the repeated slabs. Surfaces were sampled with a converged ***k***-space Monkhorst–Pack grid of 3 × 3 × 1 points, maintaining the same density of points as in the bulk. Atomic charges were calculated using Bader’s analysis.^50^

**Figure 1:**
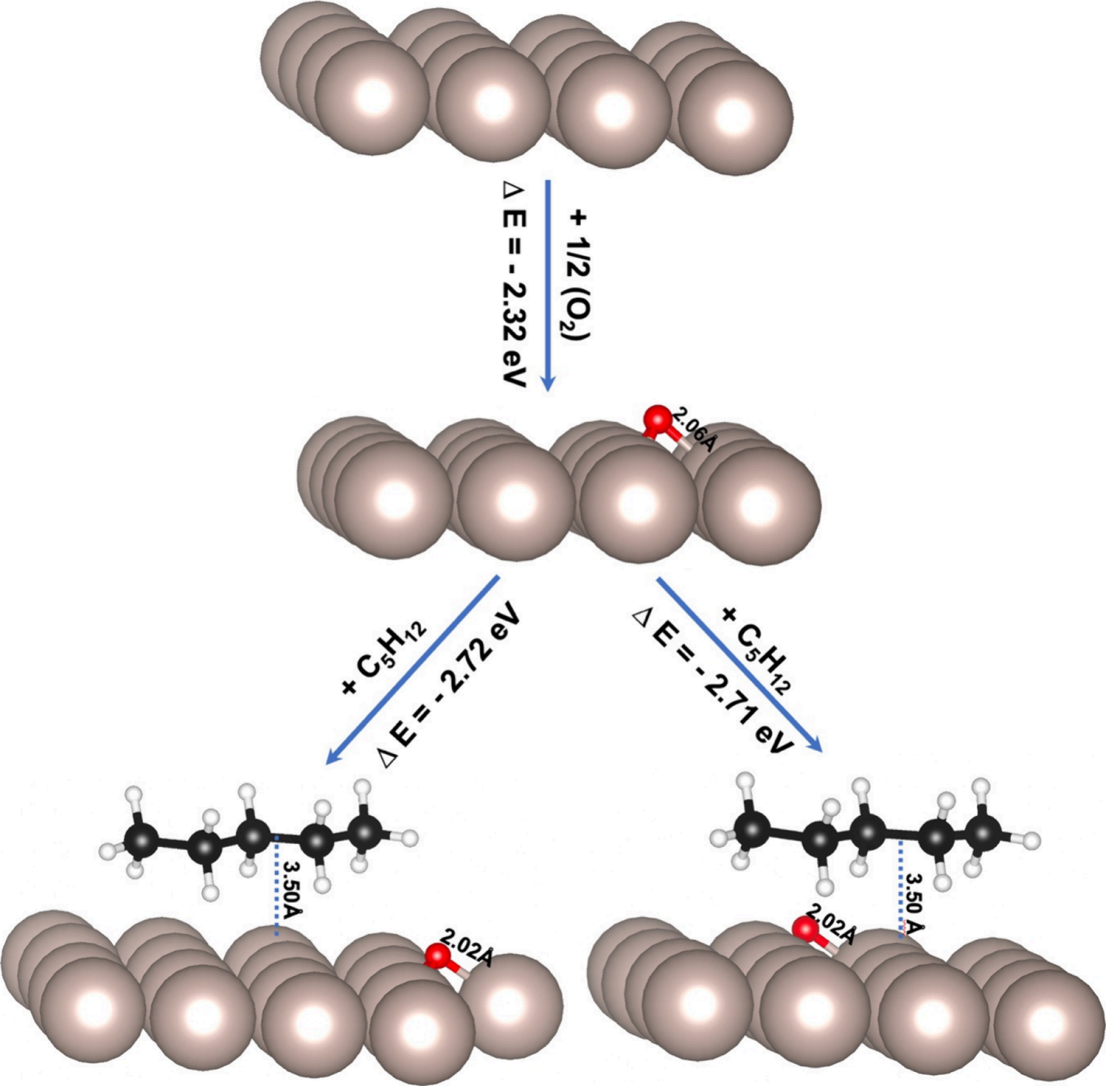
Schematic representations of oxygen adsorption at the hcp site and coadsorption of O and C_5_H_12_ on the Ru(0001) surface. The two coadsorbed figures correspond to optimized structures obtained from different starting geometries regarding the interaction of oxygen and pentane, specifically concerning carbon atoms C1 and C2, respectively. The distances (in Å) between O–H, Ru–C, and Ru–O are provided in the inset. The beige, white, red, and black spheres represent ruthenium, hydrogen, oxygen, and carbon atoms, respectively.

The initial step was to adsorb an oxygen atom on the bare surface, and then a pentane molecule was coadsorbed, as shown in Figure 1. Based on the conclusion of our previous studies,^27,28^ we have considered horizontal adsorptions of pentane on the oxidized metal surface.

The steps along the reaction pathways were characterized using the relative energy (Δ*E*) to the naked surface and gas-phase molecules (O_2_ and pentane) according to eq 1, for which the energy of O_2_ is required. Note that DFT has a well-known limitation of O_2_ overbinding.^51,52^ DFT suffers from a poor description of the electronic correlation in the d orbitals of transition metal cations.^51^ Based on a wide range of transition metal oxides, Wang et al. have proposed an overbinding correction of −1.36 eV per O_2_.^51^ Therefore, in eq 1, we added −0.68 eV to correct the oxygen atom upon calculating the relative energies along the reaction pathways, as summarized in Table 1.
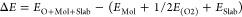
1

**Table 1:** Relative Energy (Δ*E* in eV) of Each State along the Oxidative Dehydrogenation and Partial Oxidation of Pentane at Either the Terminal (C1) or Intermediate (C2) Carbons^a^

	**Δ***E***(eV)**	
	**without O overbinding**	**with O overbinding**(51)
adsorption of O at O_hcp_	–2.32	–3.00
adsorption of O at O_fcc_	–2.00	–2.68
adsorption of O + pentane	–2.72	–3.40
intermediate formation		
C1	–1.94	–2.62
C2	–1.91	–2.59
pentene + water formation		
C1	–1.45	–2.13
C2	–1.51	–2.19
alcohol formation		
C1	–1.74	–2.42
C2	–1.85	–2.53
desorption of pentene		
C1	1.29	
C2	1.24	
desorption of alcohol		
C1	–0.69	
C2	–1.11	

a Energies are presented without and with correction of the O_2_ over-binding energy according to ref (51).

The transition states search along the reaction pathway was conducted using the climbing-image Nudged-Elastic-Band (cNEB) and Dimer methods implemented in VASP.^53^ Vibrational analyses of all optimized geometries were performed to verify local minima and saddle-points character. The activation energies (*E*_A_) were calculated as the difference between the transition state energy (*E*_TS_) and initial state energy (*E*_IS_) for the forward reaction (eq 2 and reported in Table 2).
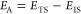
2

**Table 2:** Comparison of Calculated Activation Energy *E*_A_ (eV) for Intermediate, Pentene, and Alcohol Formation Either through C1 or C2

	***E***_**A**_**(eV)**	
	**C1**	**C2**
intermediate formation	1.81	1.97
pentene formation	1.64	1.35
alcohol formation	2.01	1.62

## 3 Results and Discussion

### 3.1 Adsorption of Oxygen and Pentane on Ru(0001)

The balance between catalytically active sites and available oxygen is important in ensuring partial oxidation instead of complete oxidation, forming CO_*x*_ compounds. Therefore, the most critical factor in the selective oxidative catalysis of hydrocarbons is the specificity of the active site to limit the amount of surface-adsorbed oxygen available at the reaction site.^54^ The nature of adsorbed oxygen should be nucleophilic (selective oxidation) rather than electrophilic (total oxidation).^54,55^

The adsorption of oxygen on a Ru(0001) surface has been extensively studied both experimentally^56−58^ and theoretically.^59−61^ Between the two on-surface 3-fold sites, hcp- (O_hcp_) and fcc-hollow (O_fcc_), the O_hcp_ site is thermodynamically preferred.^60,61^ Our calculated and overbinding-corrected adsorption energies at the O_fcc_ and O_hcp_ are −2.68 and −3.00 eV, respectively (Table 1). These align with previously calculated values of −2.47 eV for O_fcc_ and −2.96 eV for O_hcp,_ respectively,^60^ but are slightly underestimated compared to the experimental value of −3.47 eV.^56^ The calculated O–Ru bond distance is 2.06 Å, also in agreement with the experimental bond distance (2.01 ± 0.06 Å).^62^

Pentane was found to be physisorbed on pristine Ru(0001) with an adsorption energy of −0.71 eV and at a distance to the metal surface, *d*_C–Ru_*,* of 3.10 Å.^27,28^ The pentane coadsorption on the already oxygenated metal surface has an adsorption energy of −0.40 eV, depicted in Figure 1. The lack of significant molecular distortion and distance between pentane and the oxygenated metal surface (e.g., *d*_C–Ru_ = 3.50 Å and *d*_H–Ru_ = 1.34 Å) suggests the physisorption of pentane, is similar to that on pristine Ru(0001) (Figure 1). However, the difference in the calculated adsorption energies and the bond distances to the surface indicates that the oxygen acts as a slight repulsive force for the pentane.

### 3.2 Pentane First Dehydrogenation

**Figure 2:**
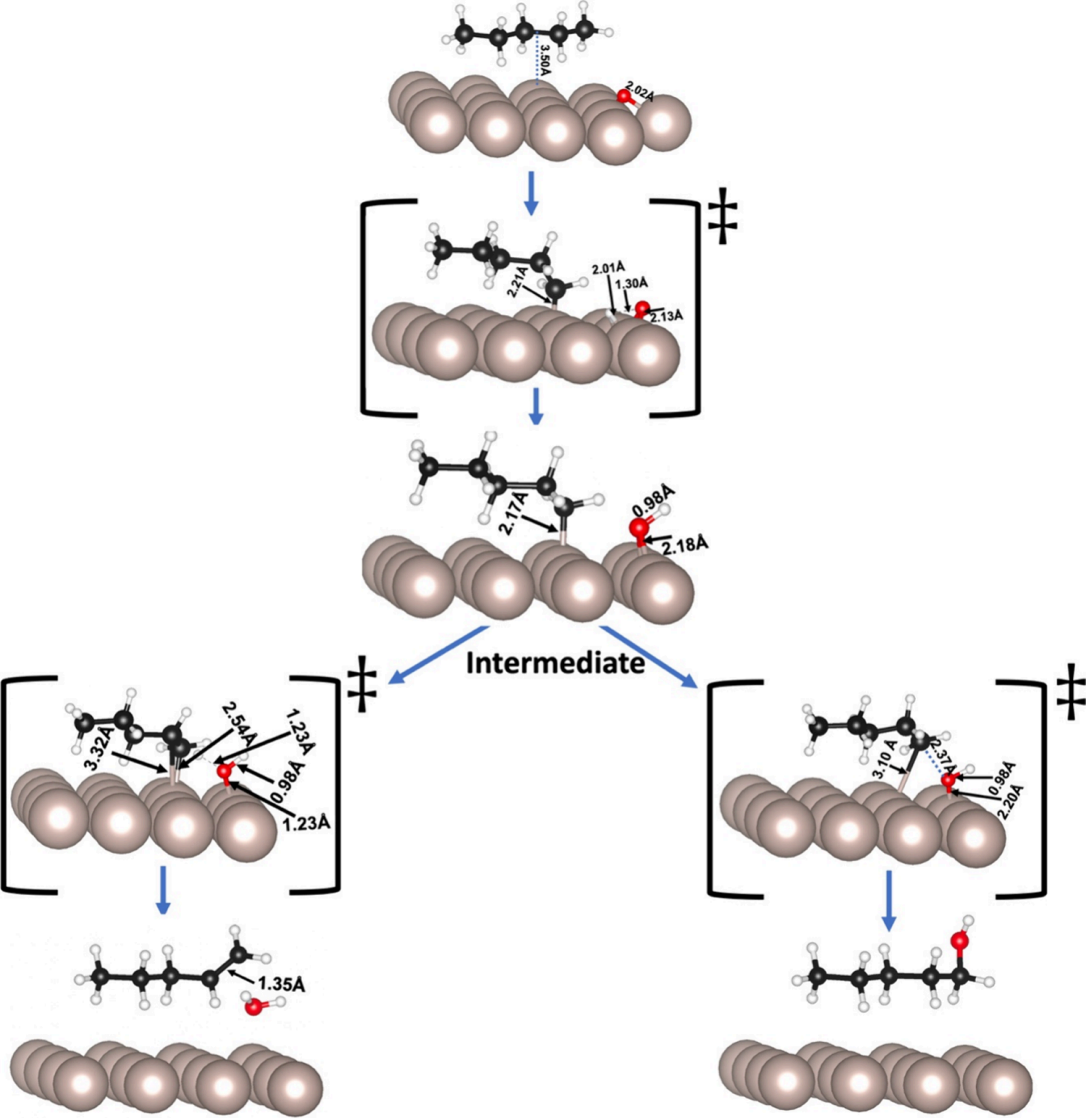
Schematic representations of partial oxidation of C1 in pentane on the O/Ru(0001) surface, including the transition states. The inset provides key distances (in Å) between Ru–C, Ru–O, and O–H. The beige, white, red, and black spheres represent ruthenium, hydrogen, oxygen, and carbon atoms, respectively.

**Figure 3:**
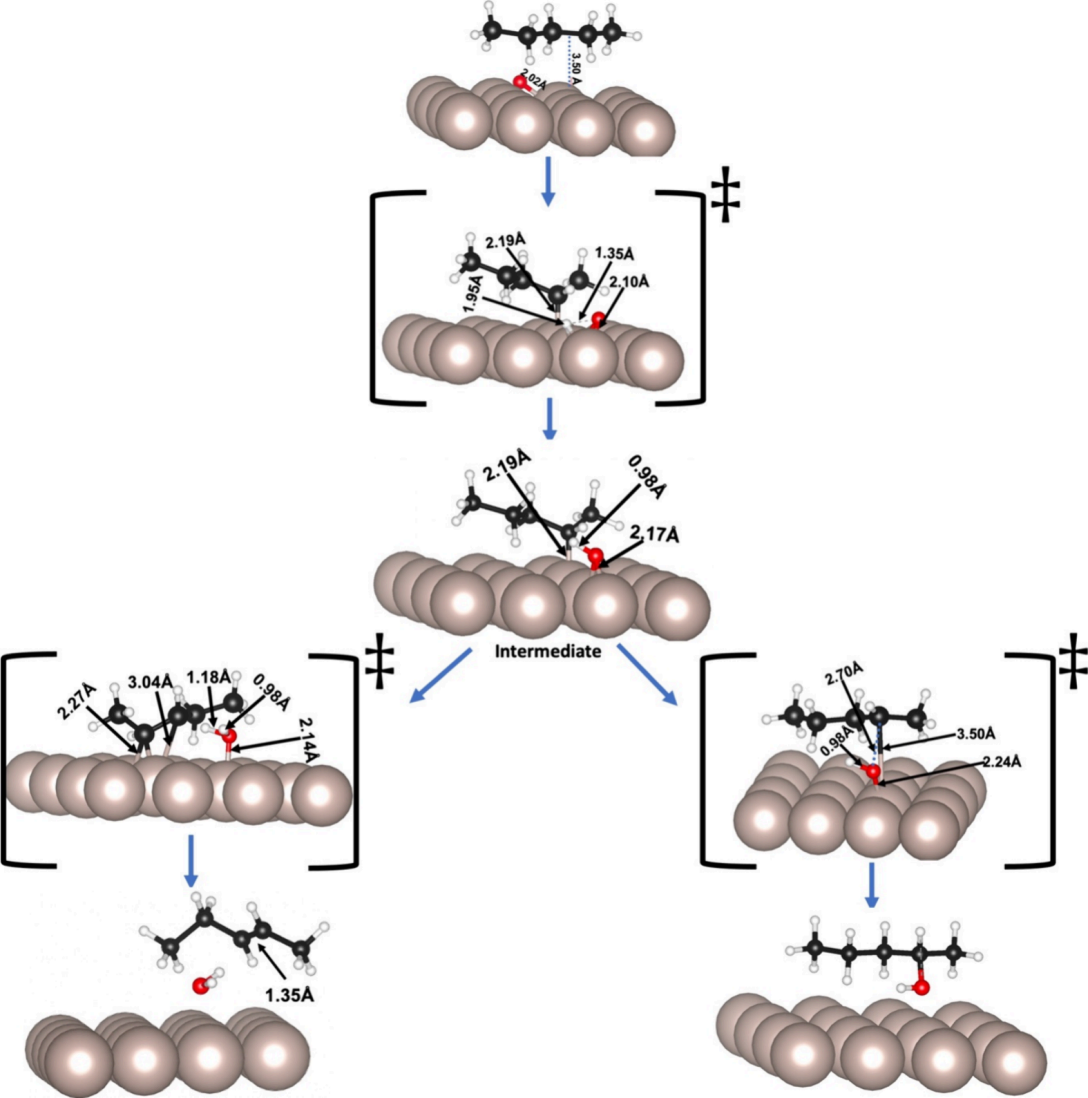
Schematic representations of partial oxidation of C2 in pentane on the Ru(0001) surface, including the transition states. The distances (in Å) between Ru–C, Ru–O, and O–H are provided in the inset. The beige, white, red, and black spheres represent ruthenium, hydrogen, oxygen, and carbon atoms, respectively.

The partial oxidation of alkanes encompasses the C–H bond activation and oxidation through the nucleophilic surface oxygen.^4,63^ We simulated the C–H cleavage either at the terminal (C1, Figure 2) or intermediate (C2, Figure 3) carbons, which led to the formation of the intermediate structure, *i.e.* coadsorbed pentyl hydride and OH (reaction R1).
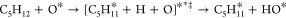
R1

The calculated relative energies for the dissociative adsorption of pentane on O/Ru(0001) and formation of the intermediate structure through C1 (−2.62 eV) is slightly more favorable than through C2 (−2.59 eV) (Table 1). This variation is reflected in the optimized interatomic bonding as the *d*_C–Ru_ at C1 (2.17 Å) is slightly shorter than that at C2 (2.19 Å).

To get a deeper insight into the process, we calculated the activation energies for the formation of the intermediate structures derived from the dehydrogenation of C1 (*E*_A_ = 1.81 eV) and C2 (*E*_A_ = 1.97 eV) (Table 2). The transition state was confirmed by careful investigation of vibrational modes. In the transition state structure, abstracted hydrogen stays at a distance to the coadsorbed oxygen of *d*_H–O_ = 1.30 Å, near the Ru surface (*d*_H–Ru_ = 2.01 Å). The final structure is the coadsorbed pentyl hydride and hydroxyl groups on the ruthenium surface (intermediate structures in Figures 2 and 3, respectively, for C1 and C2 processes). In both cases, the calculated Bader atomic charges show that the pentyl hydride and the hydroxyl accumulate, respectively, 0.2 and 0.5 e from the surface (Table 3), similar to the direct dehydrogenation of alkanes.^27,64^

**Table 3:** Relative Energy (Δ*E* in eV) for the Desorption of Pentene and Alcohol at Either the Terminal (C1) or Intermediate (C2) Carbon

	**Δ*E* (eV)**
	**without O overbinding**
desorption of pentene	
C1	1.29
C2	1.24
desorption of alcohol	
C1	–0.69
C2	–1.11

**Figure 4:**
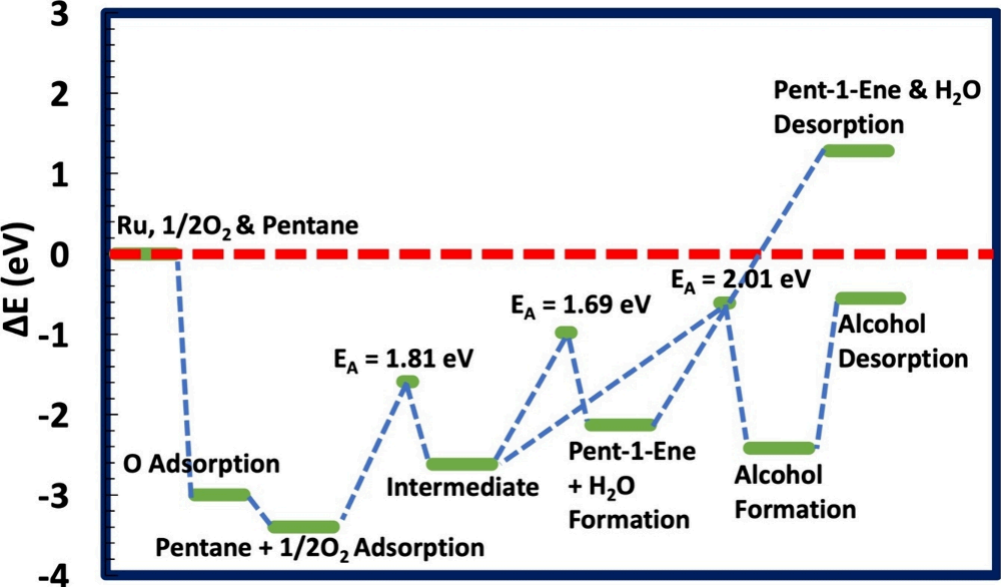
Energy profiles for forming pent-1-ene and pentan-1-ol from pentane’s C1 and an oxygenated Ru(0001). The activation energy (*E*_A_) values for all the processes are depicted. The dashed red line indicates the energy reference.

**Figure 5:**
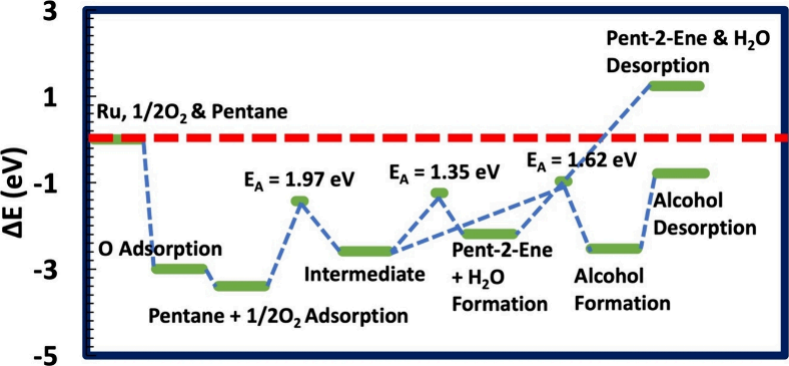
Energy profiles for forming pent-2-ene and pentan-2-ol from pentane’s C2 and an oxygenated Ru(0001). The activation energy (*E*_A_) values for all the processes are depicted. The dashed red line indicates the energy reference.

Compared with the nonoxidative direct dehydrogenation (DDH), the oxidative dehydrogenation (ODH) is thermodynamically favorable. It can be operated under mild conditions, improving energy efficiency and catalyst stability while offering pathways for selectivity.^65,66^ In DDH and ODH, the weakly physisorbed state first undergoes C–H bond activation and dehydrogenation on its way to a more strongly chemisorbed state; the pentyl hydride formation in ODH (Δ*E* = −2.62 eV) is more favorable than in DDH (Δ*E* = −0.60 eV^27^). However, the DDH activation energies for the first direct dehydrogenation of pentane on the Ru(0001) surface range between 0.78 and 0.98 eV,^27^ more accessible than through the ODH (*E*_A_ = 1.81–1.97 eV) (Table 2 and Figures 4 and 5). The significant ODH energy barrier is associated with the impact of oxygen adatom in the alkane vicinity, hindering the donation of electronic density to form the C–Ru bond.

### 3.3 Pentene Formation

An alkene can be formed through a successive second dehydrogenation of the pentyl hydride, where the participating carbon atoms are reduced from a sp^3^ hybridization to a sp^2^, forming a C=C double bond upon desorption.^64^ The surface hydroxyl pulls the second hydrogen driven by the formation of water. Water evolution was also observed for the ODH of ethane on Rh^67^ and pentane on metal-oxide catalysts.^68,69^ The ODH reaction through C1–C2 and C2–C3 leads to pent-1-ene (Δ*E* = −2.13 eV) and the preferable pent-2-ene (Δ*E* = −2.19 eV) alongside H_2_O formation (Figures 2 and 3). A significant advantage of ODH of alkanes over DDH (Δ*E* > −0.47 eV^27^) is the thermodynamic drive for forming water instead of surface-adsorbed hydrogens. Forming pent-2-ene is more favorable than pent-1-ene in both the ODH (by −0.06 eV) and DDH (by −0.16 eV^27^) mechanisms.

The analysis of the ODH transition state (Figures 2 and 3) shows the formation of pentene dihydride coadsorbed with an H moving toward the OH* group on the surface (reaction R2) at an activation energy of 1.64 and 1.35 eV for C1 and C2 respectively. The dehydrogenated carbons in the transition state lie almost parallel to the metal surface, giving rise to a di-σ-mode of adsorption with a C–C distance of ∼1.42 Å, between a single and a double bond.^70^ The calculated Bader’s charge showed that the transition state acquires −0.2 e and −0.6 e from the Ru surface, mainly centered on the C and O, respectively. The abstracted second hydrogen atom interacts with the hydroxide group to form a water molecule. Finally, the pentene and water molecules desorb from the metal surface (reaction R3).
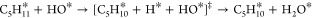
R2
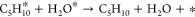
R3

The desorption of water and pent-1-ene or pent-2-ene from the metal surfaces requires 1.29 and 1.24 eV, respectively, with respect to the isolated pentane and oxygen (Table 3 and Figures 4 and 5). These results align with the desorption of pent-1-ene and pent-2-ene (1.22 and 1.13 eV, respectively) from a hydrogenated ruthenium surface.^27^ A slightly higher desorption energy for ODH than DDH (0.07–0.11 eV) shows the oxophilicity of the Ru surface, which agrees with a previous theoretical study.^23^ In general, the formation of pentene through ODH mechanism along the reaction profiles (Figures 4 and 5) follows:

a) Oxygen chemisorbs on the Ru(0001) surface (Step 1), while pentane physisorbs on the oxygenated surface through long-range interaction (Step 2).
b) Activation of pentane on the oxygenated surface occurs through (i) C–H cleavage and chemisorption of pentyl hydride and abstraction of hydrogen by surface oxygen to form the hydroxyl group (Step 3; reaction R1). Thus, it yields a coadsorbed pentyl hydride and hydroxyl groups on the Ru(0001), which serves as the intermediate structure for partial oxidation.
c) Oxidative dehydrogenation (ODH) of pentyl hydride intermediate yields pentene together with water through a second C–H dissociation to create chemisorbed pentene dihydride and the abstraction of hydrogen by the hydroxyl group to form H–O–H species on the metal surface (Step 4; reaction R2). Finally, pentene and water molecules desorb from the surface (Step 5; reaction R3).

### 3.4 Pentanol Formation

The proposed mechanism for the catalytic partial oxidation of *n*-C_5_H_12_ involves the coadsorbed pentyl hydride and hydroxyl group, enhancing the production of oxygenated compounds, especially C_5_H_11_OH.^37^ The formation of pentanol follows a similar pathway to the pentene formation except in the last two reaction steps (reactions R2 and R3) at the right-hand-side pathways in Figures 2 and 3.

We investigated the interaction of hydroxyl with the unsaturated carbon atoms, terminal (C1) and second carbon (C2). In particular, we focused on the association of adsorbates in which HO–Ru and C–Ru bonds break to form HO–C bond, leading to pentyl alcohol (reaction R4). In this process, C1 and C2 yield pentane-1-ol and pentane-2-ol, respectively, with relative energies of −2.42 and −2.53 eV (Table 1). The activation energies for these steps are 2.01 and 1.62 eV (Table 2). These values show that, like olefin production by the ODH mechanism, the alcohol formation in C2 is more favorable than in C1. The origin of the more significant barrier for pentan-1-ol compared to pentan-2-ol could be associated with the shorter C–Ru, O–Ru, and O–C bond distances of the former case (*d*_C1–Ru_ = 3.10 Å < *d*_C2–Ru_ = 3.50 Å, *d*_O–Ru_ (C1) = 2.20 Å < *d*_O–Ru_ (C2) = 2.24 Å, and *d*_O–C1_ = 2.37 Å < *d*_O–C2_ = 2.70 Å). This result is reasonable as the bond strength of the primary C–H bond (420 kJ/mol) is more significant than the secondary C–H bond (401 kJ/mol).^11^ The analysis of the charge distribution indicates that the carbons and oxygen atoms attached to the surface gain ∼0.1 e and ∼0.5 e from the metalic surface (Table S1).
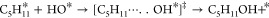
R4

The final reaction step shows the desorption of the alcohol, which, contrarily to pentene, is thermodynamically favorable with respect to isolated reactants, *i.e.*, lies below the reference energy (Table 3).

Based on the investigation of the reaction energies and activation barriers along the pathways, we have observed that the partial oxidation of pentane on the ruthenium surface may lead to pentene and pentanol. In both cases, the calculated activation energy values are significant and more prominent than through the DDH mechanism. This is because, once the surface is oxygenated, the oxygen pulls electrons from the metal surface, hindering the formation of pentyl hydride and pentene dihydride, which also pull electrons from the metal surfaces to stabilize the C–Ru bonds.

In general, the activation energy for the formation of pentanol is ∼0.30 eV, higher than that for pentene formation through the ODH mechanism. Still, due to the higher thermodynamic stability of pentanol compared to pentene, pentan-2-ol yield may be a significant proportion of the products. However, this output should be confirmed by, for instance, microkinetic simulations. Note that the conversion of pentane to other oxygenated products, *e.g.*, aldehyde, ketones, acids, *etc*, is possible but lies beyond the scope of this work.

## 4 Summary and Conclusions

The partial oxidation of *n*-pentane on the Ru(0001) surface has been investigated by theoretical calculations at the RPBE-D3 level of approach. The dissociative adsorption of pentane on the oxygenated surface creates a stable pentyl-hydride and hydroxyl intermediate, enabling oxidative dehydrogenation and oxygen insertion into the aliphatic molecule. The second dehydrogenation step occurs through a di-σ-mode pentene dihydride adsorption to form C=C, yielding pentene thermodynamically driven by the formation of a water molecule. The limiting activation energy is for the first dehydrogenation, hindering the reaction kinetics. Based on the thermodynamics and kinetic factors, the formation of pent-2-ene via ODH is favorable over DDH. However, the interaction of the hydroxyl with the coadsorbed pentyl hydride, ODH intermediate, may yield pentyl alcohol. Although the alcohol formation has a slightly higher energy barrier, the final product is more likely than pentene. The calculated formation energy, activation barriers, and alcohol desorption energy indicate that forming pentan-2-ol is more favorable than pentan-1-ol and pentene. We conclude that Ru acts as a suitable candidate for the partial oxidation of alkanes to olefins and oxygenates, with a higher probability of oxygenate formation.

## Supporting Information Available

Calculated effective charge (e) on carbon, ruthenium, oxygen, and hydrogen atoms (PDF)
