# Supplementary material for: Insights into Catalytic Oxidative Reaction Mechanisms of Pentane on the Ru(0001) Surface
Source: J Phys Chem C Nanomater Interfaces. 2024 Nov 11;128(46):19621–9. doi: 10.1021/acs.jpcc.4c05735 (PMC11587082; doi:10.1021/acs.jpcc.4c05735)
Supplement: Supplementary file 1 — jp4c05735_si_001.pdf [file jp4c05735_si_001.pdf]

Supplementary Information for:

Insights into Catalytic Oxidative Reaction Mechanisms of Pentane on Ru(0001) Surface

Mazharul M. Islam, C. Richard A. Catlow and Alberto Roldan

Cardiff Catalysis Institute, School of Chemistry, University of Cardiff, Main Building, Park Pl, Cardiff CF10 3AT, Cardiff
Email: roldanmartineza@cardiff.ac.uk

Contents

1. **Effective Charge**..2

1. Effective Charge

Table S1. Calculated effective charge (e) on carbon, ruthenium, oxygen, and hydrogen atoms.

**Transition State for Intermediate Formation**	**Charge (e)**
C-Attached to Ru	-0.30
Ru-Attached to C	0.10
O	-0.86
Ru-Attached to O	0.16
Ru-Attached to O	0.16
H Attached to O	0.20
**Intermediate**	
C-Attached to Ru	-0.25
Ru-Attached to C	0.03
O	-1.08
Ru-Attached to O	0.15
Ru-Attached to O	0.16
H Attached to O	0.60
**Transition State for Pentene and Water Formation**	
C-Attached to Ru	-0.14
Ru-Attached to C	-0.01
C-Attached to Ru	-0.22
Ru-Attached to C	-0.01
O	-1.13
Ru-Attached to O	0.03
Ru-Attached to O	0.01
Ru-Attached to O	0.12
H Attached to O	0.41
H Attached to O	0.61
**Transition State for Alcohol Formation**	
C-Attached to Ru	-0.10
Ru-Attached to C	-0.06
O	-1.08
C-Attached to Ru	-0.11
Ru-Attached to O	0.17
Ru-Attached to O	0.04
Ru-Attached to O	0.02
H Attached to O	0.62
